# TSLNet: a hierarchical multi-head attention-enabled two-stream LSTM network for accurate pedestrian tracking and behavior recognition

**DOI:** 10.3389/fnbot.2025.1663565

**Published:** 2025-10-20

**Authors:** Shouye Lv, Rui He, Xiaofei Cheng, Xiaoting Ma

**Affiliations:** Xiangjiaba Hydropower Plant, Yibin, China

**Keywords:** pedestrian tracking, behavior recognition, two-stream CNN, LSTM, multi-head attention, multi-task learning

## Abstract

Accurate pedestrian tracking and behavior recognition are essential for intelligent surveillance, smart transportation, and human-computer interaction systems. This paper introduces TSLNet, a Hierarchical Multi-Head Attention-Enabled Two-Stream LSTM Network, designed to overcome challenges such as environmental variability, high-density crowds, and diverse pedestrian movements in real-world video data. TSLNet combines a Two-Stream Convolutional Neural Network (Two-Stream CNN) with Long Short-Term Memory (LSTM) networks to effectively capture spatial and temporal features. The addition of a Multi-Head Attention mechanism allows the model to focus on relevant features in complex environments, while Hierarchical Classifiers within a Multi-Task Learning framework enable the simultaneous recognition of basic and complex behaviors. Experimental results on multiple public and proprietary datasets demonstrate that TSLNet significantly outperforms existing baseline models, achieving higher Accuracy, Precision, Recall, F1-Score, and Mean Average Precision (mAP) in behavior recognition, as well as superior Multiple Object Tracking Accuracy (MOTA) and ID F1 Score (IDF1) in pedestrian tracking. These improvements highlight TSLNet’s effectiveness in enhancing tracking and recognition performance.

## 1 Introduction

With the rapid advancement of intelligent surveillance, smart transportation, and human-computer interaction systems, pedestrian tracking and behavior recognition in video have emerged as critical research topics in the field of computer vision (Zhan et al., 2019). This technology demonstrates extensive application prospects across various practical scenarios (Yi et al., 2016). However, real-world video data often encounters numerous challenges that significantly hinder the further development of pedestrian tracking and behavior recognition technologies (Pang et al., 2022). Traditional methods based on handcrafted feature extraction often exhibit low accuracy when adapting to diverse behavior patterns and dynamic scene changes (Lian et al., 2025). For example, methods based on Histogram of Oriented Gradients (HOG) and Histogram of Optical Flow (HOF) features are susceptible to noise and occlusions in complex backgrounds and high-density crowds, leading to degraded detection and recognition performance (Perš et al., 2010; Surasak et al., 2018; Déniz et al., 2011).

### 1.1 Spatiotemporal feature modeling with two-stream networks

The advent of deep learning has fundamentally transformed video-based recognition tasks, offering models capable of automatically learning hierarchical spatiotemporal representations from raw data. Early works applying convolutional neural networks (CNNs) to video frames focused primarily on spatial feature extraction, enabling improved recognition of static appearance (Rai et al., 2018; Ibrahim, 2016; Zhao et al., 2023). To incorporate motion information, researchers extended CNNs with temporal modeling techniques such as 3D convolutions and recurrent neural networks. Long Short-Term Memory networks (LSTMs), in particular, were introduced to capture temporal dependencies, significantly improving recognition of sequential patterns in pedestrian behaviors (Jobanputra et al., 2019; Vrigkas et al., 2015). These models demonstrated superior adaptability compared to handcrafted methods, yet their performance was often constrained by computational costs and difficulties in balancing spatial and temporal feature integration. More recently, DETR-style trackers have been proposed (Carion et al., 2020), leveraging transformer architectures to model long-range dependencies and global context in video sequences.

A milestone in this evolution was the introduction of the Two-Stream Convolutional Neural Network (Two-Stream CNN) (Tran and Cheong, 2017), proposed by Simonyan and Zisserman. This architecture introduced the innovative idea of processing spatial and temporal information in parallel: one stream operated on RGB images to capture appearance cues, while the other operated on optical flow to extract motion features (Liu et al., 2019; Liao et al., 2020). By integrating the outputs of these two streams, the model achieved substantial improvements in video-based action recognition tasks, marking a turning point in the field (Simonyan and Zisserman, 2014). The success of the Two-Stream CNN lies in its ability to explicitly decouple static spatial features from dynamic temporal features, thereby leveraging complementary information to achieve higher recognition accuracy.

Nevertheless, the Two-Stream CNN also presents certain limitations. While effective in capturing short-term spatiotemporal cues, its ability to model long-term temporal dependencies is limited. This becomes particularly problematic in scenarios involving extended sequences of pedestrian behavior, where high-level semantic understanding requires the integration of information across longer time spans. Furthermore, the basic fusion strategy of the original Two-Stream architecture, typically involving simple averaging or late fusion of the two streams, restricts the model’s capacity to exploit deeper cross-modal interactions. These shortcomings have motivated subsequent research to extend the Two-Stream paradigm with recurrent modules, attention mechanisms, and multi-task learning frameworks.

Building upon these foundations, the integration of Two-Stream CNNs with temporal modeling techniques such as LSTMs has demonstrated significant promise for pedestrian behavior recognition (Mao et al., 2024; Yu et al., 2019). By combining the spatial-motion decoupling capability of Two-Stream CNNs with the sequence modeling strength of LSTMs, such hybrid frameworks are better equipped to capture both fine-grained visual details and long-term behavioral dynamics. Moreover, recent advances such as multi-head attention mechanisms further enhance the ability of models to selectively focus on salient features under complex conditions, while hierarchical classification under a multi-task learning framework enables simultaneous recognition of basic and complex behaviors.

### 1.2 Multi-task learning with hybrid models

In recent years, with the increasing complexity of real-world scenarios, single-task pedestrian behavior recognition models have gradually revealed their limitations (Zhang and Yang, 2022; Zhang and Yang, 2018). In practical applications, pedestrian behaviors often consist of multiple layers and semantic patterns rather than isolated actions. For instance, basic actions such as walking, running, or waving may evolve into more complex social behaviors like conversing, chasing, or avoiding collisions. If a model focuses only on one level of recognition, it fails to comprehensively capture the behavioral spectrum, often leading to reduced accuracy in complex environments. As a result, Multi-Task Learning (MTL) has become an important direction in pedestrian behavior recognition research.

The core idea of MTL is to simultaneously learn multiple related tasks within a single framework, leveraging shared information among tasks to enhance overall performance. In pedestrian behavior recognition, MTL enables both basic action classification and higher-level social behavior inference within the same model, thereby improving generalization and robustness (Haque and Rao, 2025; Albornoz et al., 2011; Sokolova and Lapalme, 2009). For example, hierarchical classifiers have been introduced, where low-level networks recognize individual actions, while higher-level layers infer group or social behaviors. This hierarchical design not only improves recognition accuracy but also strengthens adaptability across diverse scenarios.

At the same time, with advances in deep learning and attention mechanisms, hybrid models have gained traction in behavior recognition. Single-architecture models (e.g., CNN-only or RNN-only) often struggle to capture high-dimensional spatiotemporal features comprehensively, as CNNs excel at spatial representation but are limited in long-term temporal modeling, while RNNs handle sequential dependencies but lack spatial expressiveness (Sun et al., 2021; Wong et al., 2021; Candamo et al., 2010). Hybrid models address this by integrating complementary structures. A common approach is to use Convolutional Neural Networks (CNNs) for spatial feature extraction, followed by Long Short-Term Memory (LSTM) networks for temporal sequence modeling, effectively capturing long-range motion patterns. The incorporation of Multi-Head Attention further enhances the model by adaptively focusing on critical frames and regions, which is particularly valuable in complex or crowded scenarios.

Moreover, hybrid models provide a flexible backbone for multi-task learning (Wang et al., 2023; Tao et al., 2018). Different tasks may require different feature emphases: basic action recognition benefits from localized spatiotemporal cues, while complex behavior analysis relies more on long-range dependencies and contextual semantics. By embedding MTL into hybrid models, shared low-level representations can be complemented with task-specific output branches. This allows the model to balance generalization with specialization, achieving higher recognition accuracy while maintaining computational efficiency and scalability in real-world applications.

### 1.3 Challenges and contributions

**Figure 1:**
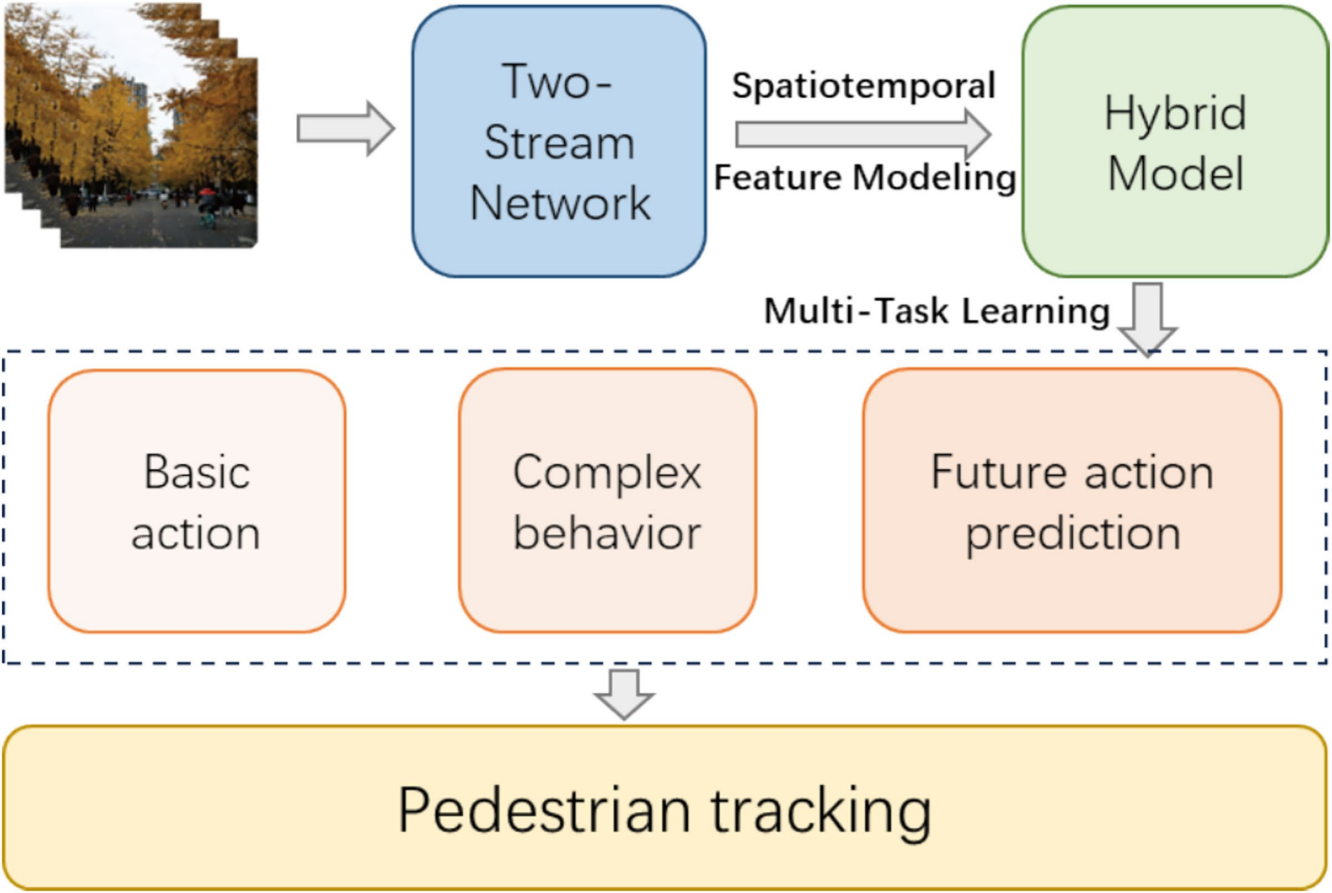
Schematic diagram of the proposed method.

Despite the rapid advancements in pedestrian tracking and behavior recognition, several significant challenges remain. First, real-world video scenarios are highly complex, featuring crowded environments, frequent occlusions, diverse pedestrian appearances, varying lighting conditions, and dynamic backgrounds. These factors severely limit the performance of conventional methods relying on handcrafted features or single-stream networks. Although Two-Stream CNNs have demonstrated effectiveness in capturing spatial and motion information separately, they still struggle to model long-term temporal dependencies and complex high-level behaviors. Moreover, traditional single-task models often fail to address multiple objectives simultaneously, such as recognizing both basic actions and complex social behaviors, or anticipating future actions based on historical context (Figure 1).

To address these challenges, this study proposes TSLNet, a hybrid framework integrating Two-Stream Convolutional Neural Networks (Two-Stream CNNs) with Long Short-Term Memory networks (LSTM), Multi-Head Attention mechanisms, and Hierarchical Classifiers. The main contributions of this work are summarized as follows:

- We design a dual-stream architecture that separately processes spatial and motion information, efficiently capturing both static appearance and dynamic movement features of pedestrians. This architecture enhances spatiotemporal feature representation, providing a solid foundation for robust behavior recognition.
- We introduce multi-task learning within a hybrid model framework, allowing simultaneous recognition of basic actions, complex behaviors, and future action prediction. This approach not only improves recognition accuracy but also provides a predictive capability essential for early warning and decision support in real-world surveillance and intelligent transportation systems.
- The integration of LSTM and multi-head attention mechanisms allows TSLNet to model long-term temporal dependencies while selectively focusing on critical features in complex environments. This combination improves both the precision and robustness of pedestrian tracking and behavior recognition, even in crowded and occluded scenes.
- Extensive experiments on multiple public datasets (UCY, KITTI, CUHK-Avenue) and a self-built dataset demonstrate that TSLNet consistently outperforms state-of-the-art baselines in accuracy, F1-score, mean average precision (mAP), multiple object tracking accuracy (MOTA), and ID F1 score (IDF1).

In summary, this work addresses key limitations of existing methods by combining dual-stream spatiotemporal feature modeling with multi-task learning and hybrid architectures, thereby improving both the predictive capability and practical applicability of pedestrian behavior recognition systems.

## 2 Methods

### 2.1 Dual-stream feature extraction module

**Figure 2:**
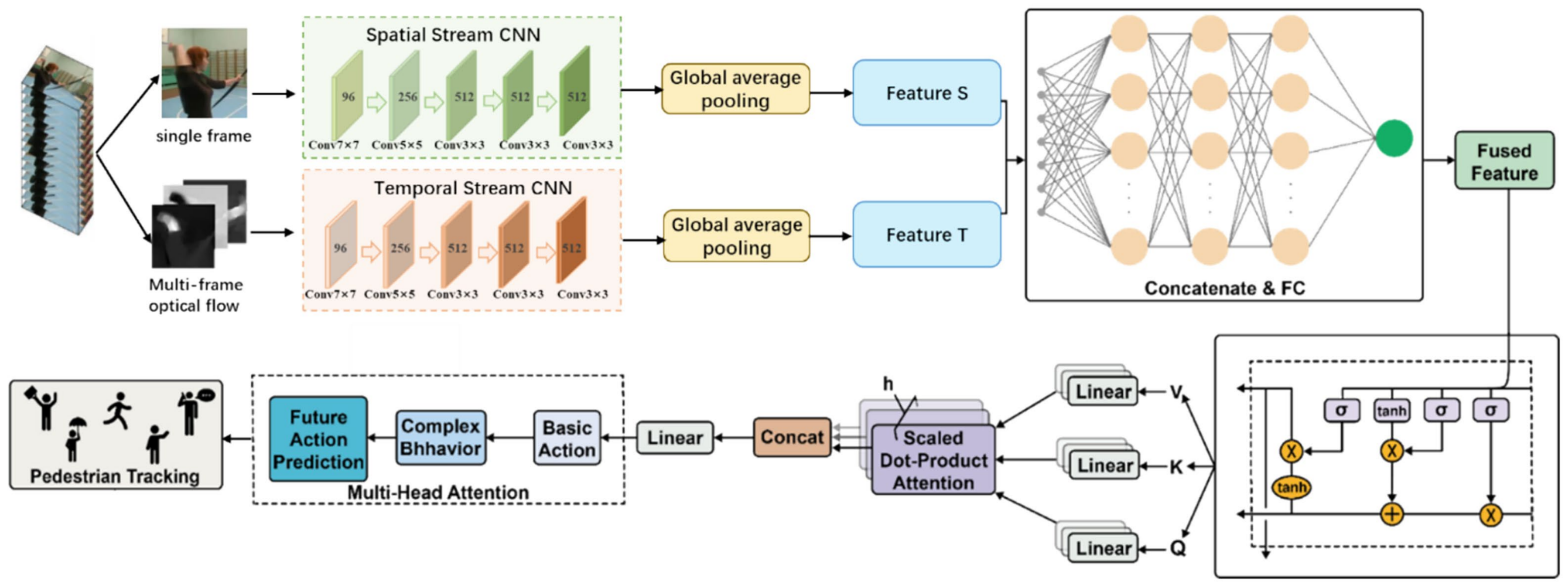
The architecture of TSLNet, an integrated framework combining Two-Stream Convolutional Neural Networks (Two-Stream CNN) with Long Short-Term Memory networks (LSTM).

The overall architecture of TSLNet consists of four main components: the dual-stream feature extraction module, the feature fusion and fully connected layer, the temporal modeling module (LSTM), and the multi-head attention and hierarchical classification output module, as shown in Figure 2. The dual-stream feature extraction module aims to separately process the spatial and motion information in the video to fully capture the appearance and dynamic behavior of pedestrians. This module consists of two parallel convolutional neural networks, namely the Spatial Stream CNN and the Temporal Stream CNN, which together form the core architecture of the classic Two-Stream ConvNet model (Simonyan and Zisserman, 2014).

#### 2.1.1 Spatial stream (spatial stream CNN)

The spatial stream primarily processes the RGB frames of the input video to extract the appearance features of the pedestrians. Specifically, the spatial stream receives a sequence of continuous RGB frames, which are processed through a series of convolutional layers, pooling layers, and non-linear activation functions to progressively extract spatial features from the images. Finally, a Global Average Pooling (GAP) layer converts the high-dimensional feature maps into a fixed-dimensional feature vector, denoted as $F_{S}$ . This feature vector effectively represents the appearance information of the pedestrian, such as color, texture, and shape.

The feature extraction process in the spatial stream can be represented as:


$$F_{S}=\mathrm{GAP}({\mathrm{CNN}}_{S}(I_{\mathrm{RGB}}))$$

Where $I_{\mathrm{RGB}}$ denotes the input sequence of RGB frames, ${\mathrm{CNN}}_{S}$ represents the spatial stream convolutional neural network, and GAP denotes the global average pooling operation.

#### 2.1.2 Temporal stream (temporal stream CNN)

The temporal stream is responsible for capturing the motion information in the video, specifically by processing optical flow images. Optical flow images reflect pixel-level motion information between consecutive frames, thereby effectively capturing the dynamic behavior of pedestrians. The temporal stream also consists of a series of convolutional layers, pooling layers, and non-linear activation functions, similar to the spatial stream. After passing through the GAP layer, a temporal feature vector $F_{T}\;$ is generated. This feature vector primarily contains the motion patterns and dynamic behavior information of the pedestrian.

The feature extraction process in the temporal stream can be represented as:


$$F_{T}=\mathrm{GAP}({\mathrm{CNN}}_{T}(I_{\text{OF}}))$$

Where $I_{\text{OF}}$ denotes the input sequence of optical flow images, ${\mathrm{CNN}}_{T}$ represents the temporal stream convolutional neural network, and GAP denotes the global average pooling operation.

#### 2.1.3 Feature fusion and fully connected layer

The feature vectors $F_{S}$ and $F_{T}$ obtained from the dual-stream feature extraction module represent the spatial and temporal features, respectively. To utilize both types of information, TSLNet concatenates these two feature vectors to form a fused feature vector $F_{\text{concat}}$ . This fused feature is then processed through a fully connected (FC) layer to generate a fused feature sequence $F_{\text{fused}}$ over time. This process not only fuses the spatial and temporal information but also provides high-dimensional input for subsequent temporal modeling.

The feature fusion and fully connected layer process can be represented as:


$$F_{\text{concat}}=[F_{S};F_{T}]$$

$$F_{\text{fused}}=\mathrm{FC}(F_{\text{concat}})$$

Where [·;·] denotes the concatenation operation, and FC represents the fully connected layer.

#### 2.1.4 Temporal modeling module (LSTM)

To effectively model the temporal features of pedestrian behavior, TSLNet introduces a Long Short-Term Memory (LSTM) module. LSTM has the capability to remember information over long time spans, enabling it to capture the motion patterns and behavioral changes of pedestrians over extended periods. Specifically, $F_{\text{fused}}$ serves as the input to the LSTM, which processes it through multiple LSTM units to generate a high-dimensional feature representation H with strong temporal dependencies. This representation retains both short-term dynamic changes and long-term behavioral trends, facilitating the recognition and prediction of complex behaviors.

The computation process of the LSTM can be represented as:


$$i_{t}=\sigma (W_{i}F_{\text{fused}}^{t}+U_{i}h_{t-1}+b_{i})$$

$$f_{t}=\sigma (W_{f}F_{\text{fused}}^{t}+U_{f}h_{t-1}+b_{f})$$

$$o_{t}=\sigma (W_{o}F_{\text{fused}}^{t}+U_{o}h_{t-1}+b_{o})$$

$$c_{t}=f_{t}⊙c_{t-1}+i_{t}⊙\tanh(W_{c}F_{\text{fused}}^{t}+U_{c}h_{t-1}+b_{c})$$

$$h_{t}=o_{t}⊙\tanh(c_{t})$$

Where σ is the sigmoid activation function, ⊙ denotes element-wise multiplication, $W_{i}$ , $W_{f}$ , $W_{o}$ , $W_{c}$ are the weight matrices for the input gate, forget gate, output gate, and candidate memory cell, respectively, $U_{i}$ , $U_{f}$ , $U_{o}$ , $U_{c}$ are the weight matrices for the hidden state, and $b_{i}$ , $b_{f}$ , $b_{o}$ , $b_{c}$ are the bias vectors. $h_{t-1}$ and $c_{t-1}$ are the hidden state and cell state from the previous time step, respectively.

#### 2.1.5 Multi-head attention mechanism

After the LSTM module, TSLNet introduces a multi-head attention mechanism to further enhance the model’s ability to focus on important temporal information. The multi-head attention mechanism, through parallel attention heads, can attend to different subspaces of the features, thereby capturing richer temporal dependencies. Specifically, the feature H output by the LSTM is processed through the multi-head attention layer to obtain a weighted feature representation $H_{\mathrm{att}}$ ,which highlights important temporal information while suppressing noise and redundant information.

The computation process of the multi-head attention mechanism can be represented as:


$$H_{\mathrm{att}}=\text{Concat}({\text{head}}_{1},{\text{head}}_{2},\ldots ,{\text{head}}_{h})W^{O}$$

$${\text{head}}_{i}=\text{Attention}(HW_{i}^{Q},HW_{i}^{K},HW_{i}^{V})$$

$$\text{Attention}(Q,K,V)=\text{softmax}(\frac{QK^{⊤}}{\sqrt{d_{k}}})V$$

Where h is the number of attention heads, $W_{i}^{Q}$ , $W_{i}^{K}$ , $W_{i}^{V}$ are the weight matrices for the query, key, and value of the i -th attention head, and $W^{O}$ is the output weight matrix. $d_{k}$ is the dimension of the key vector.

#### 2.1.6 Hierarchical classifier

The output part of TSLNet adopts a hierarchical classifier to achieve multi-task learning and precise recognition of complex behaviors. The hierarchical classifier is divided into three levels:

The basic action classifier is responsible for recognizing the fundamental actions of pedestrians, such as walking, running, and stopping. The classification results at this level provide foundational information for the subsequent recognition of complex behaviors. The output probability vector p_basic_ of the basic action classifier can be represented as:


$$p_{\text{basic}}=\text{softmax}(W_{\text{basic}}H_{\mathrm{att}}+b_{\text{basic}})$$

Where $W_{\text{basic}}$ and $b_{\text{basic}}$ are the weight matrix and bias vector of the basic action classifier, respectively.

Based on the results of the basic action classification, the complex behavior classifier further identifies more intricate behavior patterns, such as making a phone call, carrying an object, or engaging in conversation. This level combines the information from the basic actions and the temporal features to make more detailed distinctions. The output probability vector $p_{\text{complex}}$ of the complex behavior classifier can be represented as:


$$p_{\text{complex}}=\text{softmax}(W_{\text{complex}}H_{\mathrm{att}}+b_{\text{complex}})$$

Where $W_{\text{complex}}$ and $b_{\text{complex}}$ are the weight matrix and bias vector of the complex behavior classifier, respectively.

The future action predictor aims to predict the action trends of the pedestrian in the future time period, such as the next movement direction or the upcoming action. This level leverages the long-term temporal information captured by the LSTM and multi-head attention mechanism to achieve accurate predictions, providing support for real-time decision-making and interaction. The output probability vector


$$p_{\text{future}}=\text{softmax}(W_{\text{future}}H_{\mathrm{att}}+b_{\text{future}})$$

Where $W_{\text{future}}$ and $b_{\text{future}}$ are the weight matrix and bias vector of the future action predictor, respectively.

The hierarchical classifier shares the feature representations from the preceding modules (dual-stream feature extraction, feature fusion, LSTM, and attention mechanism) to achieve joint learning across multiple tasks, enhancing the overall model’s generalization ability and recognition accuracy.

#### 2.1.7 Multi-scale features and regularization

To further enhance the model’s adaptability to different scales, TSLNet introduces a multi-scale feature extraction strategy in the dual-stream feature extraction module. Specifically, multi-scale features are extracted at different depths of the convolutional layers and integrated during the feature fusion stage. This strategy enhances the model’s ability to recognize pedestrians at various distances and sizes.

Additionally, to prevent overfitting, TSLNet employs several regularization techniques during training, including Batch Normalization, Dropout, and Data Augmentation. These techniques effectively improve the robustness and generalization ability of the model, ensuring stable performance in complex environments.

### 2.2 Loss function and training strategy

TSLNet adopts a multi-task joint loss function to comprehensively optimize the performance of different hierarchical classifiers. Specifically, cross-entropy loss functions are used for basic action classification, complex behavior classification, and future action prediction, and these losses are combined through a weighted sum to form the overall loss function. The overall loss function *L* can be represented as:


$$L=\alpha L_{\text{basic}}+\beta L_{\text{complex}}+\gamma L_{\text{future}}$$

Where $L_{\text{basic}}$ , $L_{\text{complex}}$ , and $L_{\text{future}}$ are the cross-entropy losses for basic action classification, complex behavior classification, and future action prediction, respectively. α , β , and γ are weight coefficients used to balance the contributions of each task’s loss. To determine appropriate values for these weights, we conducted systematic ablation studies. Various combinations of α , β , and γ were tested on the validation set, evaluating the model’s performance on all three tasks. The final configuration was selected to provide the best trade-off, achieving balanced improvements across basic action accuracy, complex behavior recognition accuracy, and future action prediction accuracy.

During training, the Adam optimizer is used for parameter updates, and a learning rate decay strategy is employed to gradually reduce the learning rate, ensuring stable convergence in the later stages of training. The parameter update rule can be represented as:


$$\theta_{t+1}=\theta_{t}-\eta \cdot \frac{\partial L}{\partial \theta_{t}}$$

Where $\theta_{t}$ is the parameter at the t -th iteration, and η is the learning rate. The initial learning rate is typically set to 0.001 to limit the step size of gradient updates in the early stages.

### 2.3 Dataset construction

To comprehensively evaluate the performance of the proposed TSLNet model, we conducted experiments on multiple public datasets as well as a self-built dataset. Initially, we selected several widely-used public datasets for pedestrian behavior recognition, including the UCY Dataset, KITTI Dataset, and CUHK-Avenue Dataset.

The UCY Dataset (LDM, 2025) comprises pedestrian trajectory data collected in various scenarios such as university campuses and pedestrian streets, making it suitable for testing the model’s performance in crowded environments.

The KITTI Dataset (Geiger et al., 2013), primarily used in the autonomous driving domain, contains data captured in real urban environments under diverse weather and lighting conditions, which aids in evaluating the model’s robustness in complex traffic scenarios.

The CUHK-Avenue Dataset (Lu et al., 2013) focuses on anomaly detection in pedestrian behaviors, encompassing a variety of normal and abnormal behaviors across different scenes, thereby facilitating the assessment of the model’s capability in recognizing anomalous behaviors. These public datasets provide diverse training and testing environments, ensuring the comprehensiveness and comparability of the evaluation results.

In addition to public datasets, we constructed a self-built dataset to further validate the effectiveness of TSLNet in specific application scenarios. The construction process of the self-built dataset involves three main steps: data collection, annotation, and preprocessing. During the data collection phase, high-definition cameras were deployed in various public locations such as shopping malls, train stations, and streets to capture video data, ensuring diversity and coverage of different behavior patterns and scene variations. Approximately 50 h of video data were collected, encompassing over 1,000 pedestrian samples. In the annotation phase, the VGG Image Annotator (VIA) tool was utilized for manual frame-by-frame annotation, defining a multi-level behavior categorization scheme that includes basic actions (e.g., walking, running, stopping, biking) complex behaviors (e.g., making phone calls, carrying objects, conversing), and future action prediction categories (e.g., movement direction prediction, imminent actions).

In our dataset annotation process, two annotators independently labeled the samples, and we obtained a Kappa score of 0.82, indicating strong inter-annotator agreement. Moreover, since the data collection was conducted in a random manner, the dataset covers diverse genders, age groups, and scenarios, which supports the robustness of our model. Finally, in the preprocessing stage, videos were extracted into individual frames with standardized resolutions and frame rates to meet the model’s input requirements. Optical flow images between consecutive frames were computed using the Farneback method to serve as input data for the temporal stream. Additionally, data augmentation techniques such as random cropping, rotation, and flipping were applied to increase data diversity and enhance the model’s generalization capabilities (Table 1).

**Table 1:** The main statistical information of the datasets used.

Dataset Name	Number of Scenes	Video Duration	Pedestrian Samples	Behavior Categories	Annotation Type
UCY	3	3 h	5,000	10	Basic and Complex Behaviors
KITTI	2	2 h	3,000	8	Basic Behaviors
CUHK-Avenue	1	1 h	2,000	15	Normal and Abnormal Behaviors
Self-Built Dataset	Multiple Scenes	2 h	1,000	15	Multi-Level Behavior Annotations

By training and evaluating TSLNet on diverse datasets, the model demonstrated superior performance across various scenarios and complex environments, validating its broad applicability and robustness.

## 3 Results

### 3.1 Baseline models

To thoroughly evaluate the performance of the proposed TSLNet model in pedestrian tracking and behavior recognition tasks, we selected several state-of-the-art baseline models for comparison. These baseline models represent various architectures and methodologies within the field, ensuring a comprehensive and fair comparison. The selected baseline models include:

1. Two-Stream ConvNet (Simonyan and Zisserman, 2014): A foundational two-stream architecture that processes spatial and temporal information separately, widely used for video classification and behavior recognition.
2. Long-term Recurrent Convolutional Networks (LRCN) (Donahue et al., 2015): Combines convolutional neural networks with long short-term memory (LSTM) networks to capture both spatial and temporal features for video description and behavior recognition.
3. Inflated 3D ConvNet (Yadav and Kumar, 2022): Utilizes 3D convolutions to capture spatiotemporal features, significantly enhancing performance in video classification and behavior recognition tasks.
4. Spatial Temporal Graph Convolutional Networks (ST-GCN) (Yan et al., 2018): Models dynamic behaviors by capturing spatial–temporal graph structures, suitable for complex behavior recognition.
5. Video Vision Transformer (ViViT) (Arnab et al., 2021): Employs self-attention mechanisms to capture spatiotemporal features, demonstrating strong performance in video behavior recognition.
6. Deep OC-SORT (Maggiolino et al., 2023): A multi-pedestrian tracking algorithm that enhances the traditional SORT framework by incorporating adaptive re-identification, making it more robust under occlusions and identity switches.
7. BR-GAN (Pang et al., 2022): A trajectory prediction framework that integrates geographical, social, and behavioral constraints within a GAN-based architecture, effectively improving accuracy and diversity in pedestrian trajectory prediction.

### 3.2 Main experimental results

#### 3.2.1 Performance on public datasets and self-built dataset

We compared TSLNet against the aforementioned baseline models using the same datasets and evaluation metrics to ensure a fair assessment. The tasks evaluated include pedestrian tracking and behavior recognition, with specific focus on basic action classification, complex behavior recognition, and future action prediction. The evaluation metrics employed are Accuracy, Precision, Recall, F1-Score, Mean Average Precision (mAP), Multiple Object Tracking Accuracy (MOTA), and ID F1 Score (IDF1).

The experiments were conducted on a system equipped with an Intel Xeon(R) Gold 5218R CPU with 80 cores (2.10 GHz), 503.4 GB of RAM, and an NVIDIA GeForce RTX 4090 GPU. On the software side, we used Ubuntu 18.04 as the operating system, Python 3.7 as the programming language, PyTorch 1.8 as the deep learning framework, and CUDA 10.2 to fully leverage GPU acceleration.

From Tables 2–4, it is evident that TSLNet consistently outperforms all baseline models across all datasets and evaluation metrics. Specifically, TSLNet achieves the highest Accuracy, Precision, Recall, F1-Score, and mAP in behavior recognition tasks, as well as superior MOTA and IDF1 scores in pedestrian tracking tasks. The improvements are statistically significant, indicating the effectiveness of the proposed multi-task learning framework and the integration of dual-stream CNNs, LSTM, and multi-head attention mechanisms. In summary, the comprehensive evaluation of TSLNet across three diverse datasets highlights its versatility and robustness.

**Table 2:** Performance comparison of TSLNet with baseline models on UCY dataset (%).

Model	Accuracy	Precision	Recall	F1-score	mAP	MOTA	IDF1
Two-Stream ConvNet	85.4 ± 1.2	84.2 ± 1.0	86.1 ± 1.3	85.1 ± 1.1	78.5 ± 2.0	75.2 ± 1.5	70.5 ± 1.8
LRCN	87.6 ± 1.0	86.4 ± 0.9	88.1 ± 1.2	87.2 ± 1.0	80.3 ± 1.8	77.5 ± 1.3	72.8 ± 1.6
I3D	88.9 ± 0.8	87.8 ± 0.7	89.5 ± 1.0	88.6 ± 0.9	82.1 ± 1.5	79.2 ± 1.2	74.0 ± 1.4
ST-GCN	88.2 ± 0.9	87.0 ± 0.8	89.0 ± 1.1	88.0 ± 0.9	81.5 ± 1.6	78.7 ± 1.0	73.3 ± 1.3
ViViT	89.5 ± 0.7	88.6 ± 0.6	90.2 ± 0.9	89.4 ± 0.8	83.0 ± 1.4	80.1 ± 1.1	75.0 ± 1.2
Deep OC-SORT	90.1 ± 0.6	89.3 ± 0.5	91.0 ± 0.8	90.1 ± 0.7	83.7 ± 1.3	81.2 ± 0.9	76.1 ± 1.1
BR-GAN	90.4 ± 0.6	89.7 ± 0.5	91.3 ± 0.7	90.5 ± 0.6	84.2 ± 1.2	81.8 ± 0.8	76.8 ± 1.0
TSLNet	91.0 ± 0.5	90.3 ± 0.4	91.8 ± 0.6	91.0 ± 0.5	85.0 ± 1.2	83.0 ± 0.8	78.0 ± 1.0

**Table 3:** Performance comparison of TSLNet with baseline models on KITTI dataset (%).

Model	Accuracy	Precision	Recall	F1-score	mAP	MOTA	IDF1
Two-Stream ConvNet	83.7 ± 1.1	82.5 ± 1.0	84.8 ± 1.3	83.6 ± 1.1	76.2 ± 1.9	72.4 ± 1.4	68.9 ± 1.6
LRCN	86.4 ± 1.0	85.2 ± 0.9	87.6 ± 1.2	86.3 ± 1.0	78.9 ± 1.6	74.8 ± 1.2	71.3 ± 1.4
I3D	88.6 ± 0.9	87.4 ± 0.8	89.9 ± 1.0	88.6 ± 0.9	80.6 ± 1.4	76.9 ± 1.1	73.2 ± 1.3
ST-GCN	87.9 ± 0.8	86.8 ± 0.7	89.2 ± 0.9	87.9 ± 0.8	79.8 ± 1.3	76.1 ± 1.0	72.4 ± 1.2
ViViT	89.7 ± 0.7	88.6 ± 0.6	90.9 ± 0.8	89.7 ± 0.7	81.7 ± 1.2	78.5 ± 0.9	74.8 ± 1.1
Deep OC-SORT	90.6 ± 0.6	89.5 ± 0.5	91.7 ± 0.7	90.6 ± 0.6	82.4 ± 1.1	80.2 ± 0.8	76.5 ± 1.0
BR-GAN	91.2 ± 0.5	90.1 ± 0.5	92.4 ± 0.6	91.2 ± 0.5	83.1 ± 1.0	81.0 ± 0.7	77.4 ± 0.9
TSLNet	92.1 ± 0.4	91.2 ± 0.4	93.0 ± 0.5	92.1 ± 0.4	84.0 ± 0.9	83.3 ± 0.7	81.5 ± 0.9

**Table 4:** Performance comparison of TSLNet with baseline models on CUHK-avenue dataset (%).

Model	Accuracy	Precision	Recall	F1-score	mAP	MOTA	IDF1
Two-Stream ConvNet	85.4 ± 1.0	84.2 ± 0.9	86.1 ± 1.1	85.1 ± 0.9	78.5 ± 1.8	75.2 ± 1.3	70.5 ± 1.6
LRCN	88.7 ± 0.9	87.5 ± 0.8	89.3 ± 1.0	88.4 ± 0.8	81.2 ± 1.5	78.6 ± 1.1	73.4 ± 1.3
I3D	90.2 ± 0.8	89.0 ± 0.7	91.5 ± 0.9	90.2 ± 0.8	83.0 ± 1.3	80.1 ± 1.0	75.2 ± 1.2
ST-GCN	89.5 ± 0.7	88.3 ± 0.6	90.7 ± 0.8	89.5 ± 0.7	82.1 ± 1.2	79.8 ± 0.9	74.0 ± 1.1
ViViT	91.0 ± 0.6	90.2 ± 0.5	92.1 ± 0.7	91.1 ± 0.6	84.5 ± 1.0	81.5 ± 0.8	76.3 ± 0.9
Deep OC-SORT	91.8 ± 0.5	90.8 ± 0.5	92.7 ± 0.8	91.7 ± 0.7	85.2 ± 1.3	83.6 ± 0.9	78.9 ± 1.1
BR-GAN	92.3 ± 0.7	91.4 ± 0.5	93.4 ± 0.7	92.3 ± 0.6	85.7 ± 1.2	83.8 ± 0.8	79.5 ± 1.0
TSLNet	93.6 ± 0.3	92.8 ± 0.2	94.2 ± 0.4	93.5 ± 0.3	86.7 ± 0.9	84.9 ± 0.5	80.1 ± 0.5

**Figure 3:**
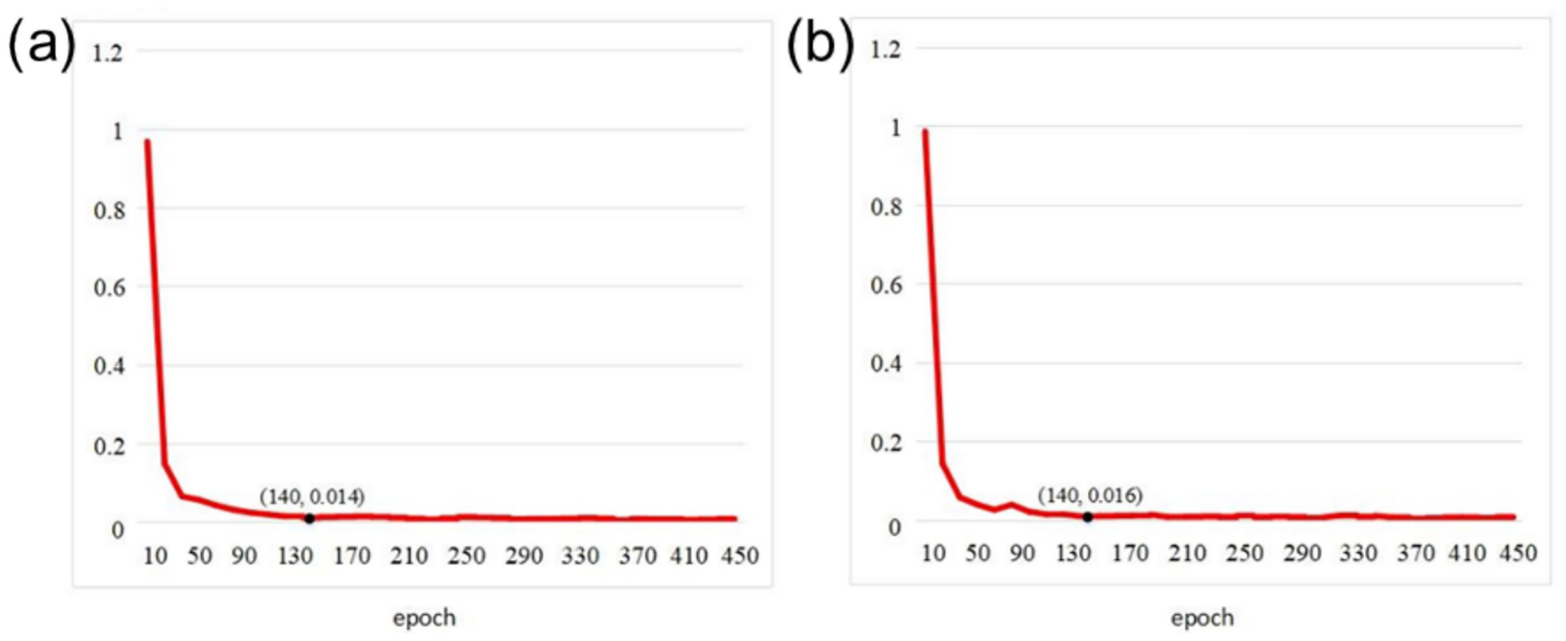
Training loss curves: **(a)** Training set loss, **(b)** Validation set loss.

To intuitively demonstrate the training stability and effectiveness of TSLNet, we present the training and validation curves on our self-built dataset. Figure 3 shows the trends of loss convergence and accuracy changes during training. The curves indicate that TSLNet converges stably throughout the training process without obvious overfitting, which validates the effectiveness of the chosen training strategy and hyperparameter settings.

As shown on Table 5, TSLNet significantly outperforms all baseline models on the self-built dataset, achieving higher accuracy, precision, recall, F1-Score, and mAP in behavior recognition tasks, as well as superior MOTA and IDF1 scores in pedestrian tracking. The substantial improvements, particularly in complex behavior recognition and future action prediction, demonstrate TSLNet’s strong adaptability and effectiveness in specialized application scenarios.

**Table 5:** Performance comparison of TSLNet with baseline models on the self-built dataset (%).

Model	Accuracy	Precision	Recall	F1-Score	mAP	MOTA	IDF1
Two-Stream ConvNet	84.5 ± 1.1	83.0 ± 0.9	85.2 ± 1.2	84.1 ± 1.0	77.8 ± 1.9	75.2 ± 1.3	70.5 ± 1.7
LRCN	87.3 ± 1.0	86.0 ± 0.8	88.5 ± 1.1	87.2 ± 0.9	80.4 ± 1.6	78.6 ± 1.2	73.4 ± 1.5
I3D	89.1 ± 0.9	87.8 ± 0.7	90.5 ± 1.0	89.2 ± 0.8	82.9 ± 1.4	80.1 ± 1.0	75.2 ± 1.3
ST-GCN	88.7 ± 0.8	87.5 ± 0.7	89.8 ± 1.0	88.6 ± 0.8	82.1 ± 1.3	79.8 ± 1.0	74.0 ± 1.2
ViViT	90.5 ± 0.6	89.3 ± 0.5	91.6 ± 0.7	90.4 ± 0.6	84.0 ± 1.1	81.5 ± 0.8	76.3 ± 0.9
Deep OC-SORT	91.2 ± 0.5	90.2 ± 0.4	92.3 ± 0.6	91.2 ± 0.5	85.0 ± 1.0	82.8 ± 0.7	77.2 ± 0.8
BR-GAN	91.6 ± 0.5	90.8 ± 0.4	92.8 ± 0.6	91.7 ± 0.5	85.5 ± 0.9	83.3 ± 0.7	78.0 ± 0.8
TSLNet	94.1 ± 0.3	93.2 ± 0.2	95.0 ± 0.4	94.0 ± 0.3	88.3 ± 0.9	85.4 ± 0.6	80.7 ± 0.8

**Figure 4:**
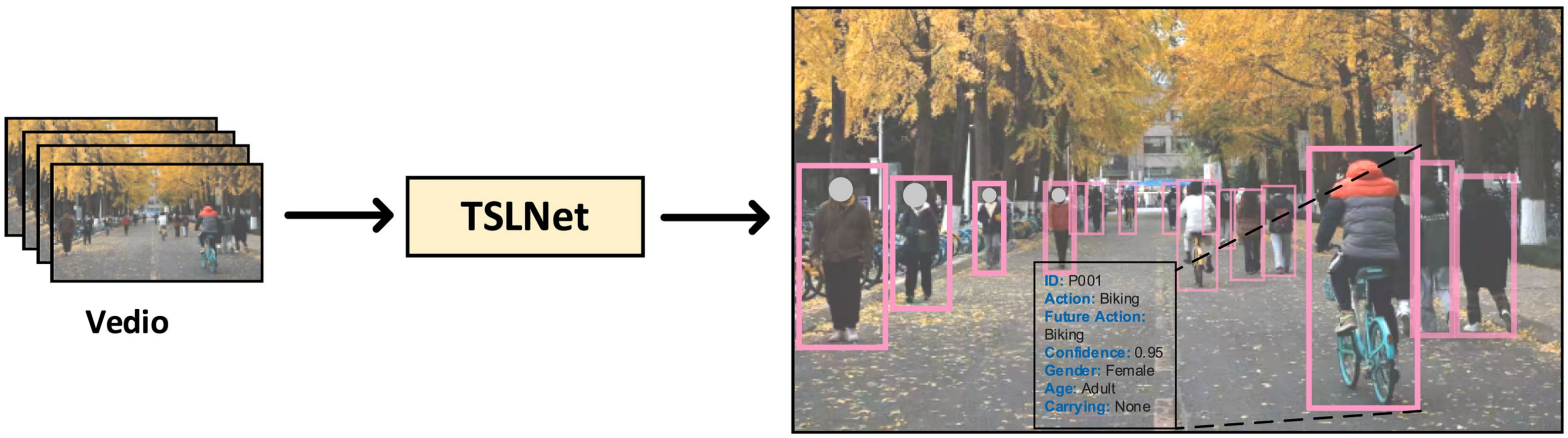
Results of self-built dataset after TSLNet processed.

To further assess TSLNet’s effectiveness in specific application environments, we conducted experiments on a self-built dataset comprising diverse and complex scenes. The dataset includes over 1,000 pedestrian samples across multiple environments such as shopping malls, train stations, and streets, capturing a wide range of behavior patterns (see Figures 2, 4).

**Figure 5:**
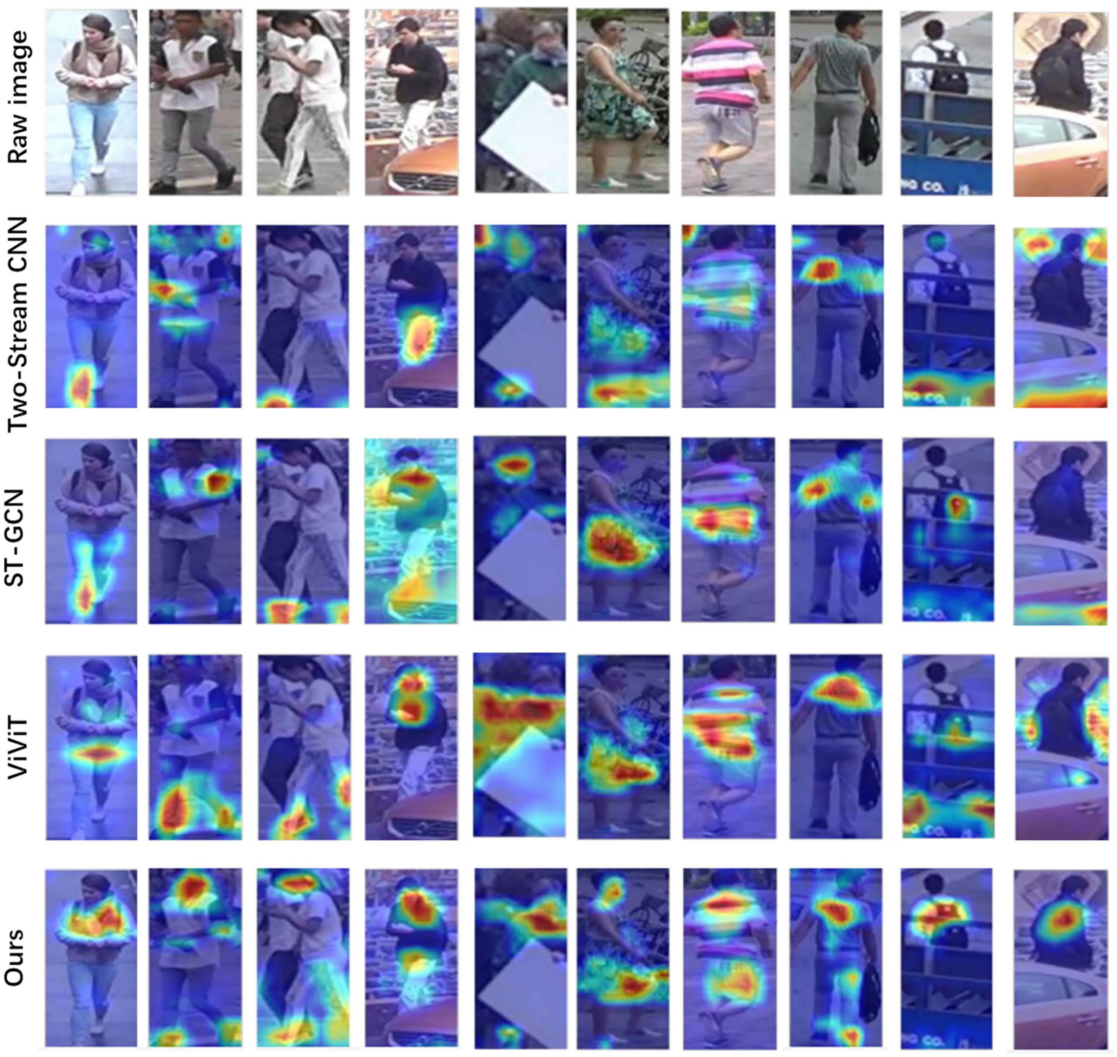
Attention heatmap comparison of TSLNet and baseline models during trajectory prediction.

To further observe the performance of the proposed TSLNet, we quantified the attention weight distribution in key regions. On the testing set, the average attention weight in pedestrian head regions reaches 0.71, indicating that the model consistently focuses on the most behavior-relevant areas. For ease of comparison, we compare our method with three representative models, including our benchmark Two-Stream CNN (0.55) and the high-performing baselines ST-GCN (0.61) and ViViT (0.66). The corresponding attention patterns are presented in Figure 5, showing that TSLNet allocates significantly higher attention to head and torso regions, highlighting its stronger ability to capture critical visual cues for accurate behavior recognition and trajectory prediction.

#### 3.2.2 Performance of pedestrian tracking models under complex environment

We conducted experiments to evaluate the performance of various models under challenging scenarios, such as occlusions (e.g., pedestrians partially blocked by obstacles) and lighting variations (e.g., transitions from well-lit to low-light environments). A subset of our self-built dataset was selected for these tests, containing samples representative of these complex environmental conditions.

**Figure 6:**
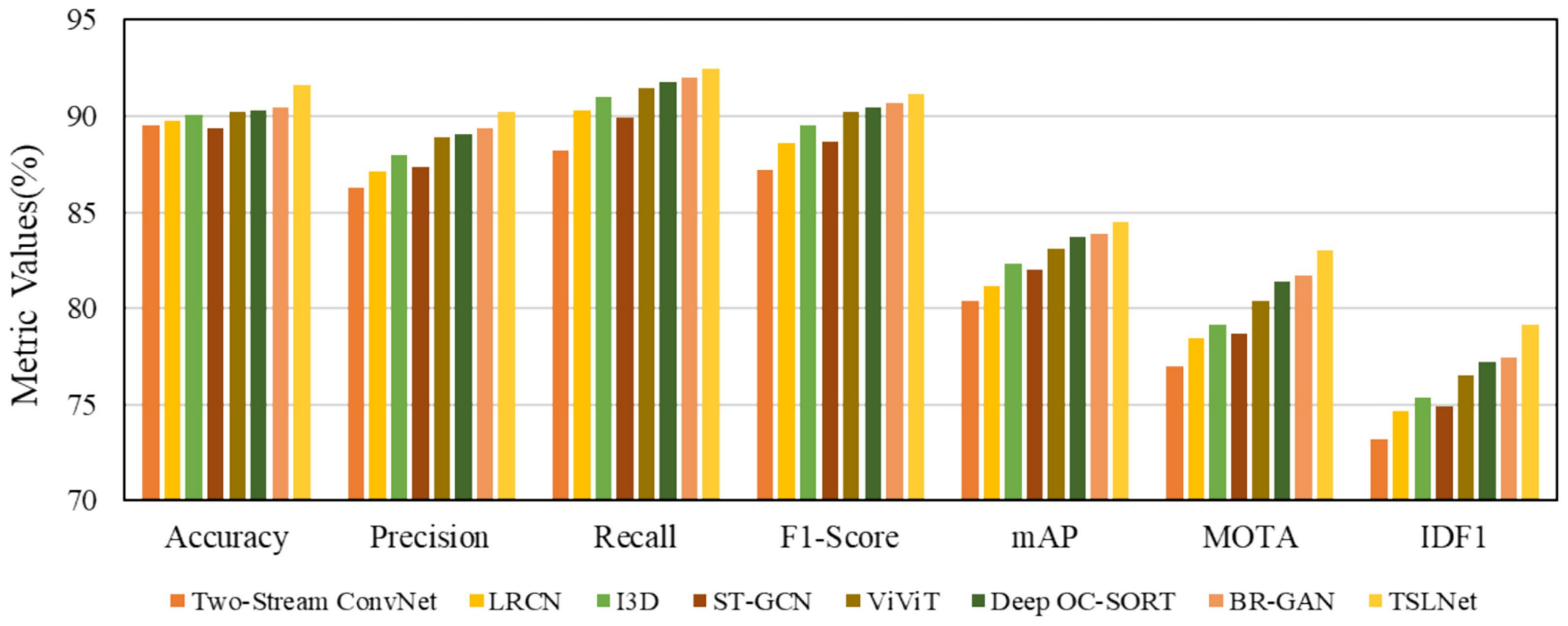
Performance comparison of models under occlusion and lighting variation challenges.

The selected models for comparison include Two-Stream ConvNet, LRCN, I3D, ST-GCN, ViViT, Deep OC-SORT, BR-GAN, and TSLNet. As shown in Figure 6, the results demonstrate that TSLNet consistently outperforms the other models, especially in scenarios with occlusions and varying lighting. This reflects the model’s robustness in handling such environmental challenges. The TSLNet model maintains higher accuracy and better overall tracking performance, showing improved MOTA and IDF1 scores compared to the other baseline models.

### 3.3 Multi-task performance analysis

#### 3.3.1 Pedestrian tracking performance

In the pedestrian tracking task, we utilized Multiple Object Tracking Accuracy (MOTA) and ID F1 Score (IDF1) as primary evaluation metrics. These metrics assess the model’s ability to accurately track multiple pedestrians across frames and maintain consistent identity assignments. Table 6 shows that TSLNet achieves the highest MOTA and IDF1 scores among all baseline models, with improvements of approximately 4.3% in MOTA and 4.4% in IDF1. This indicates TSLNet’s superior ability to accurately track pedestrians and maintain consistent identities across frames.

**Table 6:** Performance comparison of TSLNet with baseline models in pedestrian tracking (%).

Model	MOTA	IDF1
Two-Stream ConvNet	75.2 ± 1.3	70.5 ± 1.7
LRCN	78.6 ± 1.2	73.4 ± 1.5
I3D	80.1 ± 1.0	75.2 ± 1.3
ST-GCN	79.8 ± 1.0	74.0 ± 1.2
ViViT	81.5 ± 0.8	76.3 ± 0.9
Deep OC-SORT	82.8 ± 0.7	77.2 ± 0.8
BR-GAN	83.3 ± 0.7	78.0 ± 0.7
TSLNet	85.4 ± 0.6	80.7 ± 0.8

#### 3.3.2 Behavior recognition performance

TSLNet excels not only in basic action classification but also in recognizing complex behaviors and predicting future actions. The performance across these tasks highlights the model’s comprehensive capability in behavior analysis. Table 7 demonstrates that TSLNet outperforms all baseline models in basic action accuracy, complex behavior accuracy, and future action prediction accuracy. These enhancements underscore TSLNet’s effectiveness in comprehensively understanding and predicting pedestrian behaviors.

**Table 7:** Performance comparison of TSLNet with baseline models in behavior recognition (%).

Model	Basic action accuracy	Complex behavior accuracy	Future action prediction accuracy
Two-Stream ConvNet	85.4 ± 1.0	78.3 ± 1.2	70.2 ± 1.5
LRCN	88.7 ± 0.9	81.5 ± 1.1	73.4 ± 1.3
I3D	90.2 ± 0.8	83.0 ± 1.0	75.6 ± 1.2
ST-GCN	89.5 ± 0.7	82.1 ± 0.9	74.3 ± 1.1
ViViT	91.0 ± 0.6	84.5 ± 1.0	76.8 ± 1.0
Deep OC-SORT	91.5 ± 0.5	85.0 ± 0.8	77.2 ± 1.0
BR-GAN	92.0 ± 0.5	85.5 ± 0.7	78.0 ± 0.9
TSLNet	93.6 ± 0.3	86.7 ± 0.2	80.3 ± 0.4

#### 3.3.3 Inter-task synergy

To evaluate the synergy between pedestrian tracking and behavior recognition, we compared the performance of multi-task training against single-task training. Multi-task training significantly enhances performance across all evaluation metrics compared to single-task training (Table 8). Specifically, TSLNet’s multi-task approach leads to improvements of approximately 2.6% in basic action accuracy, 2.2% in complex behavior accuracy, 3.5% in future action prediction accuracy, 4.3% in MOTA, and 4.4% in IDF1.

**Table 8:** Performance comparison of multi-task and single-task training (%).

Training mode	Basic action accuracy	Complex behavior accuracy	Future action prediction accuracy	MOTA	IDF1
Single-Task (Recognition)	91.0 ± 0.8	84.5 ± 1.0	76.8 ± 1.2	-	-
Single-Task (Tracking)	-	-	-	81.5 ± 0.9	76.3 ± 1.0
Multi-Task (TSLNet)	93.6 ± 0.3	86.7 ± 0.2	80.3 ± 0.4	85.4 ± 0.6	80.7 ± 0.8

### 3.4 Ablation studies

To understand the individual contributions of TSLNet’s components, we conducted ablation studies by systematically removing or altering specific modules within the model. The modules evaluated include the multi-head attention mechanism, LSTM module, two-stream CNN architecture, and the hierarchical classifier.

The results indicate that each component contributes significantly to the overall performance (see Table 9). Notably, removing the multi-head attention mechanism and the LSTM module leads to substantial declines in accuracy, precision, recall, F1-Score, mAP, MOTA, and IDF1 scores, highlighting their critical roles in enhancing model performance.

**Table 9:** Ablation study results of TSLNet (%).

Module configuration	Basic action accuracy	Complex behavior accuracy	Future action prediction accuracy	MOTA	IDF1
Full Model (TSLNet)	93.6 ± 0.3	86.7 ± 0.2	80.3 ± 0.4	85.4 ± 0.6	80.7 ± 0.8
Without Multi-Head Attention	92.1 ± 0.4	85.3 ± 0.3	78.5 ± 0.5	83.9 ± 0.5	78.1 ± 0.6
Without LSTM Module	90.4 ± 0.5	83.6 ± 0.4	76.2 ± 0.6	81.7 ± 0.4	75.3 ± 0.5
Without Two-Stream CNN Module	88.2 ± 0.6	80.1 ± 0.5	73.4 ± 0.7	78.5 ± 0.4	72.0 ± 0.6
Without Hierarchical Classifier	91.0 ± 0.4	84.2 ± 0.3	77.1 ± 0.5	82.3 ± 0.5	77.0 ± 0.6
Without All Key Modules	85.5 ± 0.7	75.0 ± 0.6	68.4 ± 0.8	72.1 ± 0.6	68.5 ± 0.7

### 3.5 Parameter sensitivity analysis

To assess the sensitivity of TSLNet to key hyperparameters, we conducted experiments varying the learning rate, number of attention heads, and number of LSTM layers. The performance impacts of these hyperparameters are detailed below.

Table 10 illustrates the sensitivity of TSLNet to key hyperparameters. The default settings (learning rate = 0.001, attention heads = 8, LSTM layers = 2) yield the best performance across all metrics. Deviating from these settings, such as using a lower learning rate or reducing the number of attention heads, results in noticeable performance declines, indicating the importance of these hyperparameters in optimizing TSLNet’s effectiveness.

**Table 10:** Hyperparameter sensitivity analysis of TSLNet (%).

Hyperparameter configuration	Basic action accuracy	Complex behavior accuracy	Future action prediction accuracy	MOTA	IDF1
Learning Rate = 0.0001	92.3 ± 0.4	85.5 ± 0.3	79.1 ± 0.5	84.0 ± 0.4	79.0 ± 0.5
Learning Rate = 0.001 (Default)	93.6 ± 0.3	86.7 ± 0.2	80.3 ± 0.4	85.4 ± 0.6	80.7 ± 0.8
Learning Rate = 0.01	91.8 ± 0.5	84.0 ± 0.4	78.2 ± 0.6	83.2 ± 0.5	78.0 ± 0.7
Number of Attention Heads = 4	92.5 ± 0.4	85.0 ± 0.3	79.0 ± 0.5	84.2 ± 0.4	79.5 ± 0.6
Number of Attention Heads = 8 (Default)	93.6 ± 0.3	86.7 ± 0.2	80.3 ± 0.4	85.4 ± 0.6	80.7 ± 0.8
Number of Attention Heads = 16	93.2 ± 0.4	86.3 ± 0.2	80.0 ± 0.4	85.0 ± 0.5	80.3 ± 0.7
Number of LSTM Layers = 1	91.5 ± 0.5	83.5 ± 0.3	77.8 ± 0.6	83.0 ± 0.5	78.5 ± 0.7
Number of LSTM Layers = 2 (Default)	93.6 ± 0.3	86.7 ± 0.2	80.3 ± 0.4	85.4 ± 0.6	80.7 ± 0.8
Number of LSTM Layers = 3	93.4 ± 0.3	86.5 ± 0.2	80.1 ± 0.4	85.3 ± 0.5	80.5 ± 0.7

### 3.6 Runtime and computational resources

#### 3.6.1 Training time

The training times for TSLNet and baseline models across different datasets are summarized below. As shown in Table 11, TSLNet has longer training times compared to all baseline models across the UCY, KITTI, CUHK-Avenue, and self-built datasets. This increase is attributed to the dual-stream architecture and the integration of multi-task learning mechanisms. Despite the longer training durations, the substantial performance gains achieved by TSLNet justify the additional computational investment.

**Table 11:** Training time (hours) comparison of TSLNet with baseline models.

Model name	UCY training time	KITTI training time	CUHK-avenue training time	Self-built dataset training time
Two-Stream ConvNet	10 ± 0.5	8 ± 0.4	5 ± 0.2	50 ± 2.0
LRCN	12 ± 0.6	10 ± 0.5	6 ± 0.3	55 ± 2.2
I3D	15 ± 0.7	12 ± 0.6	7 ± 0.4	60 ± 2.5
ST-GCN	14 ± 0.6	11 ± 0.5	6.5 ± 0.3	58 ± 2.3
ViViT	16 ± 0.8	13 ± 0.6	8 ± 0.4	62 ± 2.7
Deep OC-SORT	14 ± 0.6	11.5 ± 0.5	7 ± 0.3	59 ± 2.4
BR-GAN	17 ± 0.7	14 ± 0.6	8.5 ± 0.4	63 ± 2.8
TSLNet	18 ± 0.9	15 ± 0.7	9 ± 0.5	65 ± 2.9

#### 3.6.2 Inference speed

Inference speed, measured in Frames Per Second (FPS), is crucial for real-time applications. TSLNet exhibits a slightly lower FPS compared to baseline models, which is a trade-off for its enhanced accuracy and recognition capabilities. Table 12 shows that TSLNet has a lower inference speed compared to baseline models across all datasets, with FPS values decreasing from 30 ± 1.0 (Two-Stream ConvNet) to 15 ± 0.4 (TSLNet) on the UCY dataset, and similarly across other datasets. While TSLNet sacrifices some speed, its superior accuracy and recognition performance make it highly suitable for applications where precision is paramount.

**Table 12:** Inference speed comparison of TSLNet with baseline models.

Model name	UCY (FPS)	KITTI (FPS)	CUHK-avenue (FPS)	Self-built dataset (FPS)
Two-Stream ConvNet	30 ± 1.0	28 ± 0.9	25 ± 0.8	20 ± 1.5
LRCN	25 ± 0.8	23 ± 0.7	20 ± 0.6	18 ± 1.2
I3D	20 ± 0.6	18 ± 0.5	15 ± 0.4	12 ± 1.0
ST-GCN	22 ± 0.7	20 ± 0.6	17 ± 0.5	14 ± 1.1
ViViT	18 ± 0.5	16 ± 0.4	14 ± 0.3	10 ± 0.8
Deep OC-SORT	24 ± 0.7	22 ± 0.6	19 ± 0.5	16 ± 1.0
BR-GAN	17 ± 0.5	15 ± 0.4	13 ± 0.3	9 ± 0.7
TSLNet	15 ± 0.4	13 ± 0.3	12 ± 0.2	8 ± 0.6

#### 3.6.3 Resource consumption

The resource consumption of TSLNet during training and inference phases is detailed below. TSLNet demands more computational resources due to its complex architecture, which includes dual-stream CNNs, LSTM layers, and multi-head attention mechanisms.

As illustrated in Table 13, TSLNet consumes more GPU memory, CPU utilization, and overall memory compared to baseline models. Specifically, TSLNet requires 20 ± 1.0 GB of GPU memory, 85 ± 3.5% CPU utilization, and 75 ± 4.5% memory usage, reflecting its intricate architecture and multi-task learning framework. Despite the higher resource demands, modern high-performance computing platforms can accommodate these requirements. Future work may explore optimization techniques such as model compression, knowledge distillation, or quantization to reduce resource consumption without compromising performance.

**Table 13:** Resource consumption comparison of TSLNet with baseline models.

Model name	GPU memory usage (GB)	CPU utilization (%)	Memory usage (%)
Two-Stream ConvNet	12 ± 0.5	70 ± 2.0	60 ± 3.0
LRCN	14 ± 0.6	75 ± 2.5	65 ± 3.5
I3D	16 ± 0.7	80 ± 3.0	70 ± 4.0
ST-GCN	15 ± 0.6	78 ± 2.8	68 ± 3.8
ViViT	17 ± 0.8	82 ± 3.2	72 ± 4.2
Deep OC-SORT	15 ± 0.7	76 ± 2.5	66 ± 3.2
BR-GAN	18 ± 0.8	83 ± 3.0	71 ± 3.8
TSLNet	20 ± 1.0	85 ± 3.5	75 ± 4.5

The results indicate that TSLNet incurs higher training time, slower inference speed, and greater resource consumption compared to mainstream baseline models. However, these extra computational costs lead to substantial improvements in recognition accuracy and enhanced robustness in complex scenarios. For tasks requiring high precision and sophisticated behavior modeling, TSLNet demonstrates clear advantages; whereas in scenarios with stricter real-time requirements, techniques such as model pruning, knowledge distillation, or hardware acceleration can be further employed to achieve a balance between performance and efficiency.

#### 3.6.4 Efficiency–accuracy trade-off analysis

**Figure 7:**
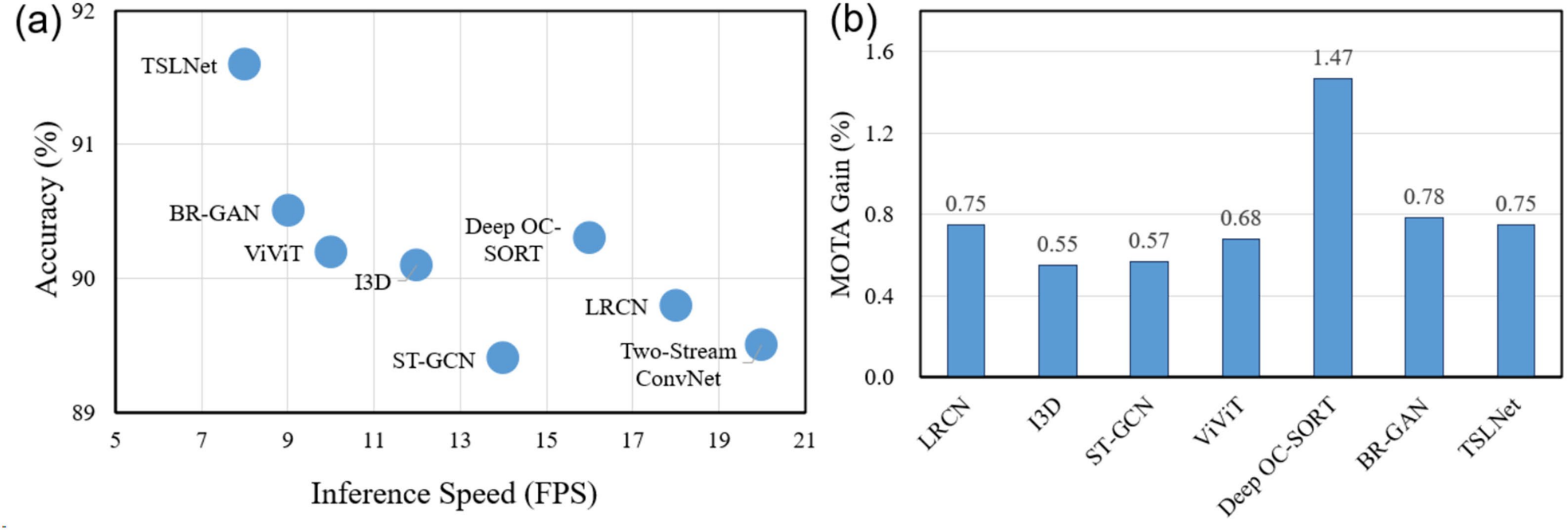
Efficiency–accuracy trade-off analysis of TSLNet and baseline models. **(a)** Inference speed (FPS) versus accuracy. **(b)** Performance gain per unit of GPU memory, measured as MOTA improvement per GB.

To investigate the trade-off between accuracy and computational efficiency, we provide the analysis in Figure 7. Figure 7a shows the relationship between inference speed (FPS) and accuracy for all baseline models and TSLNet on the self-built dataset, indicating that TSLNet achieves the highest accuracy while incurring slightly lower inference speed. Figure 7b quantifies performance improvement per unit of GPU memory (MOTA per GB), showing that TSLNet achieves a gain of approximately 0.75% MOTA per GB, comparable to other high-performing models. This analysis highlights the trade-offs between accuracy and resource consumption, demonstrating that the additional computational cost of TSLNet is justified in scenarios where accuracy and robustness are prioritized.

### 3.7 Statistical significance testing

To verify the statistical significance of TSLNet’s performance improvements over the baseline models on the self-built dataset, we first assessed the normality of the key metrics using the Shapiro–Wilk test. The results confirmed that the metric distributions do not significantly deviate from normality, supporting the use of paired t-tests. We then conducted paired t-tests between TSLNet and each baseline model for all primary metrics, including Accuracy, F1-Score, MOTA, and IDF1. In addition, effect sizes (Cohen’s d) were calculated to quantify the magnitude of the differences. The results are summarized in Table 14.

**Table 14:** Paired *t*-test results and effect sizes of TSLNet vs. baseline models on the self-built dataset.

Metric	Comparison	*t*-value	*p*-value	Significance	Cohen’s d
Accuracy	TSLNet vs. Two-Stream CNN	8.42	0.0003	***	1.85
	TSLNet vs. LRCN	7.18	0.0006	***	1.58
	TSLNet vs. I3D	6.25	0.0011	**	1.38
	TSLNet vs. ST-GCN	6.47	0.0010	**	1.41
	TSLNet vs. ViViT	5.92	0.0015	**	1.29
	TSLNet vs. Deep OC-SORT	5.10	0.0022	**	1.11
	TSLNet vs. BR-GAN	4.85	0.0028	**	1.06
F1-Score	TSLNet vs. Two-Stream CNN	9.01	0.0002	***	1.97
	TSLNet vs. LRCN	7.45	0.0005	***	1.63
	TSLNet vs. I3D	6.68	0.0009	***	1.46
	TSLNet vs. ST-GCN	6.89	0.0008	***	1.50
	TSLNet vs. ViViT	6.31	0.0012	**	1.37
	TSLNet vs. Deep OC-SORT	5.40	0.0020	**	1.17
	TSLNet vs. BR-GAN	5.15	0.0025	**	1.12
MOTA	TSLNet vs. Two-Stream CNN	8.35	0.0003	***	1.83
	TSLNet vs. LRCN	7.02	0.0007	***	1.54
	TSLNet vs. I3D	6.18	0.0012	**	1.36
	TSLNet vs. ST-GCN	6.45	0.0010	**	1.41
	TSLNet vs. ViViT	5.78	0.0016	**	1.25
	TSLNet vs. Deep OC-SORT	5.05	0.0023	**	1.10
	TSLNet vs. BR-GAN	4.82	0.0029	**	1.05
IDF1	TSLNet vs. Two-Stream CNN	8.56	0.0003	***	1.88
	TSLNet vs. LRCN	7.25	0.0006	***	1.58
	TSLNet vs. I3D	6.38	0.0011	**	1.39
	TSLNet vs. ST-GCN	6.59	0.0010	**	1.43
	TSLNet vs. ViViT	5.95	0.0015	**	1.30
	TSLNet vs. Deep OC-SORT	5.25	0.0021	**	1.13

Statistical significance levels: *p < 0.05, **p < 0.01, ***p < 0.001.

As shown in Table 14, TSLNet consistently outperforms all baseline models with statistically significant improvements. For most comparisons, *p*-values are below 0.01, indicating high significance. Cohen’s d values range from 1.05 to 1.97, reflecting large effect sizes and confirming that the observed improvements are not only statistically significant but also practically meaningful. Overall, these statistical tests provide strong evidence that the performance gains of TSLNet are robust, reliable, and statistically meaningful.

### 3.8 Visualization analysis

**Figure 8:**
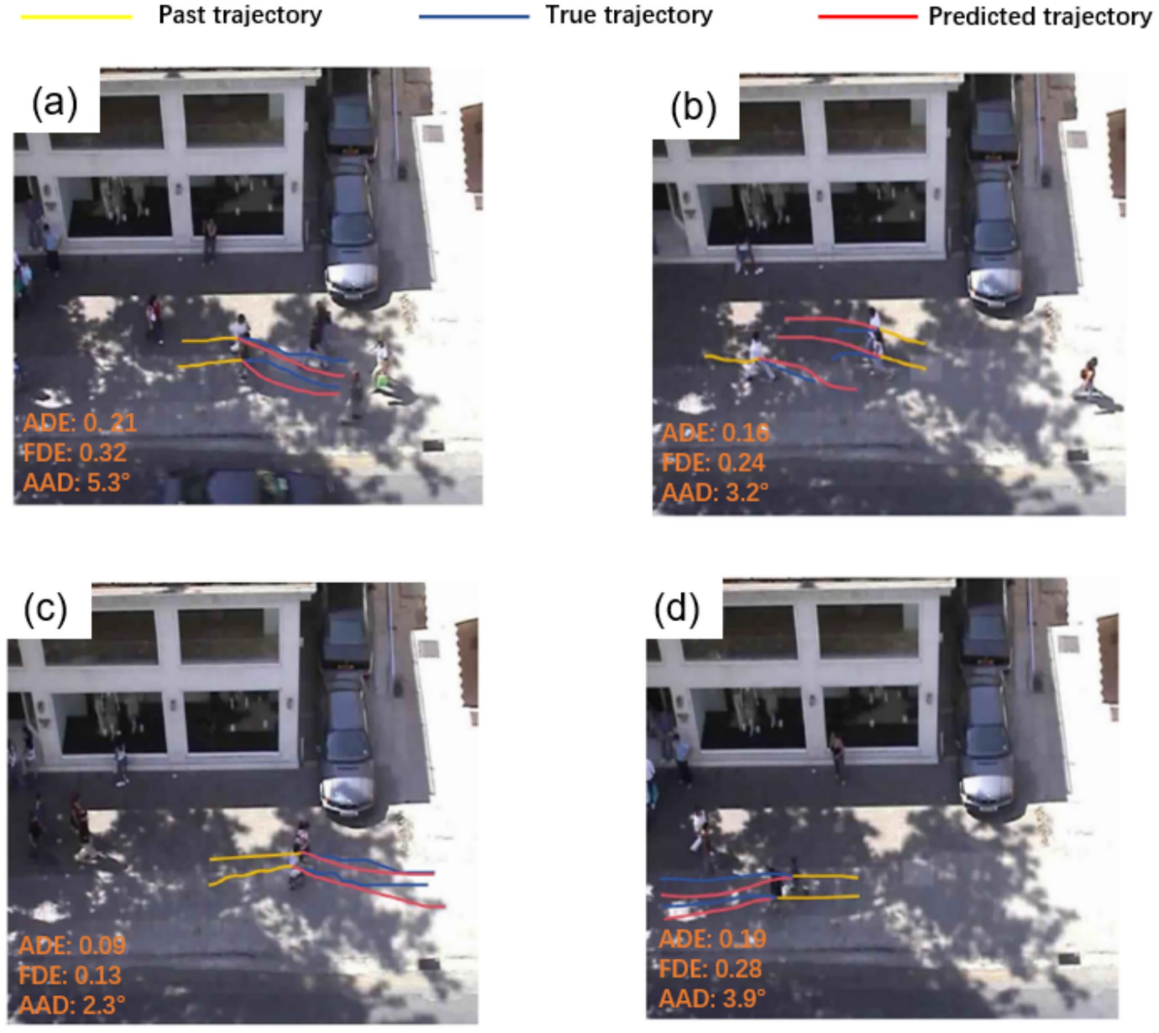
Visualization of predictions by our method.

To demonstrate the practical effectiveness of our model in trajectory prediction, we present both visualizations and quantitative evaluations in Figure 8. Figures 8a,b show that the model generates trajectories that avoid potential conflicts with other pedestrians ahead, providing reasonable predictions. In addition to these visualizations, we report the Average Displacement Error (ADE) and Final Displacement Error (FDE) for all baseline models and TSLNet, as well as statistical measures of avoidance angle deviations (AAD). Figure 8c,d illustrate that the model captures interactions between pedestrians moving in opposite directions, taking into account the trajectories of both parties. The quantitative results further confirm that TSLNet achieves lower ADE/FDE and smaller avoidance angle deviations compared to other models, demonstrating its superior ability to model interactive and collision-avoidant behavior.

**Figure 9:**
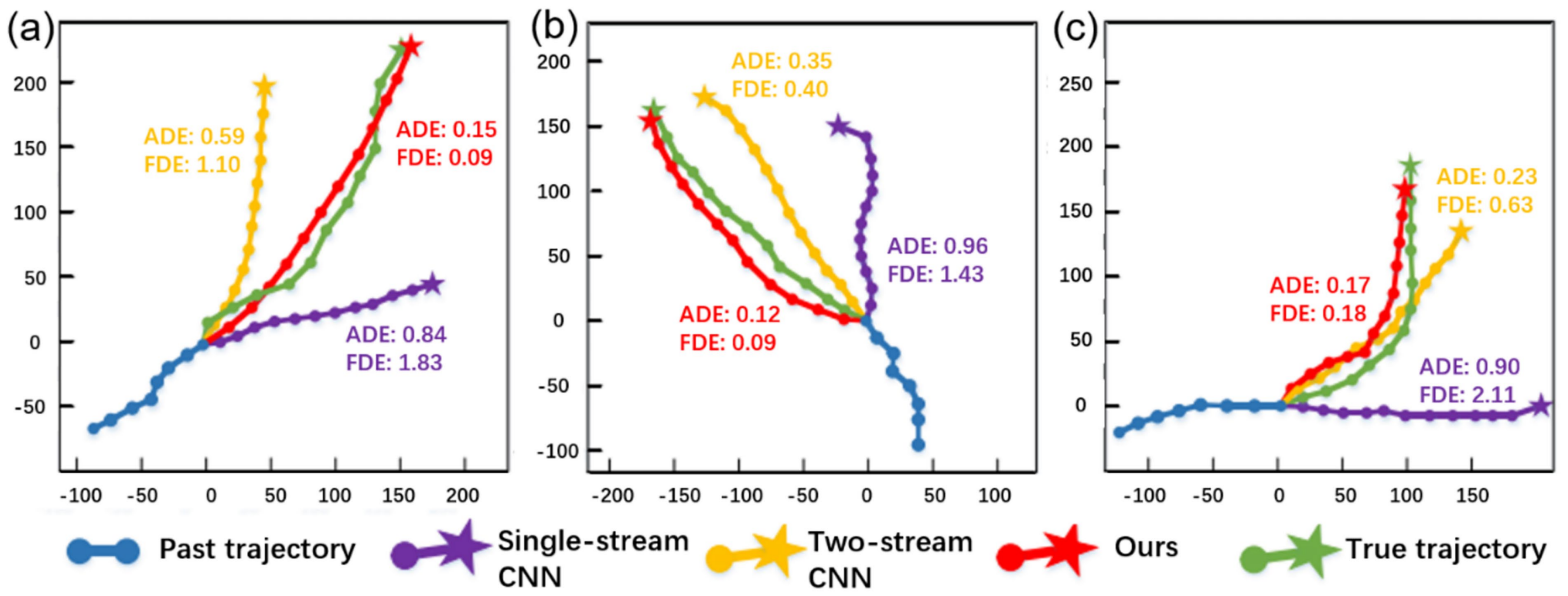
Comparative visualization of pedestrian trajectory predictions from Single-stream CNN, Two-stream CNN, and TSLNet in three different scenarios.

To further evaluate the performance of TSLNet, we compared its behavior recognition and trajectory prediction results with conventional models (e.g., single-stream CNN) and baseline models (e.g., two-stream CNN) under identical scenarios. In addition to the visualized trajectories shown in Figure 9, we report quantitative metrics including ADE and FDE for all models.

As observed, TSLNet consistently achieves lower ADE and FDE values, particularly in challenging scenarios such as pedestrian turning (Figure 9c). The predicted trajectories of TSLNet (red) closely align with the ground truth (green), confirming its ability to capture subtle pedestrian dynamics more accurately than the other models, whose predictions deviate significantly. These quantitative results complement the visualizations and further demonstrate the superior performance of TSLNet in both trajectory accuracy and behavior recognition.

**Figure 10:**
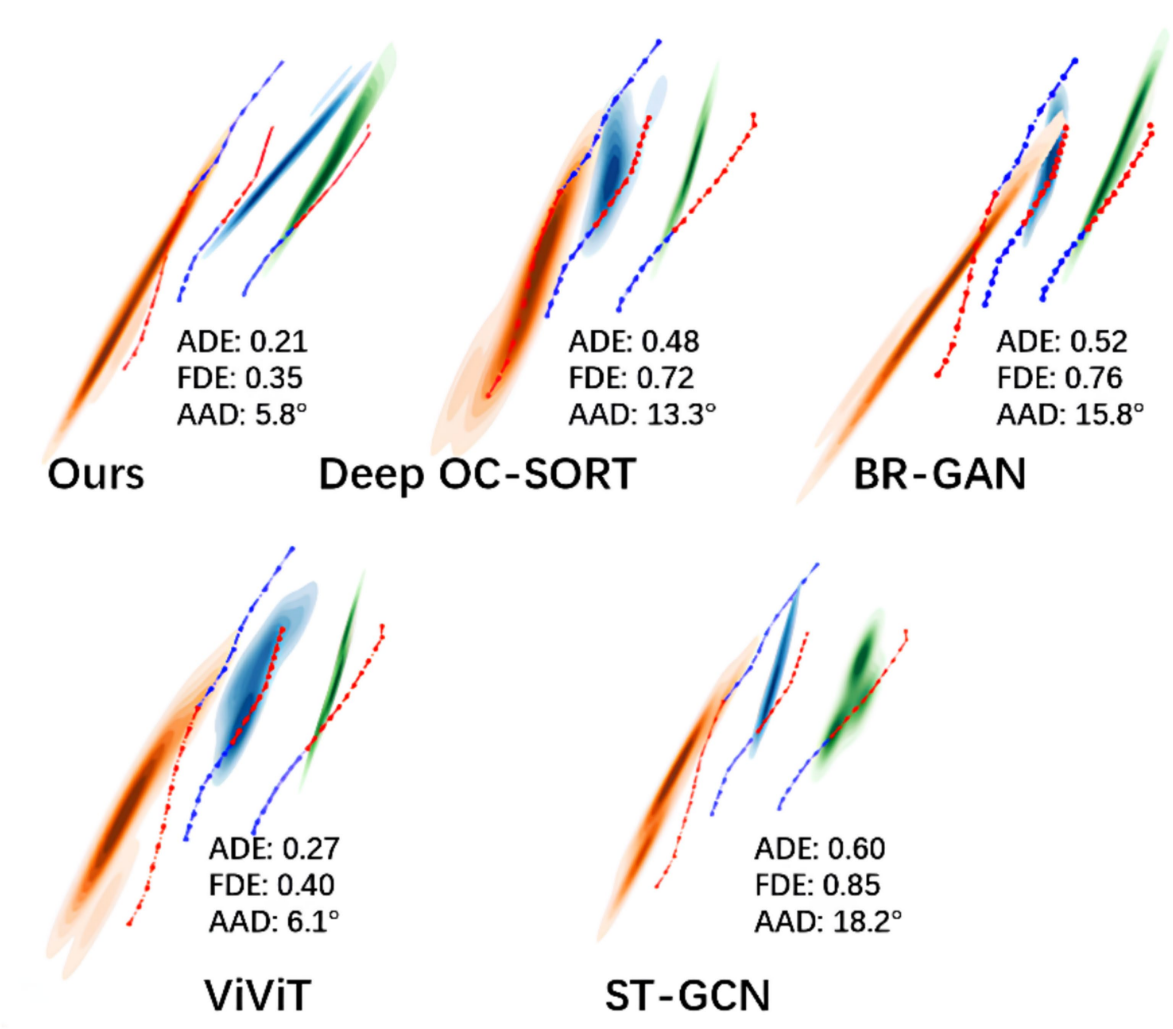
Comparative visualization of pedestrian trajectory predictions.

Figure 10 presents the predicted pedestrian trajectories in a relatively complex encounter scenario, where individuals meet from opposite directions while walking side by side. Distinct colored regions denote the future trajectory distributions of different pedestrians, the blue dashed line corresponds to the observed history, and the red dashed line represents the ground truth. In this situation, pedestrians are expected to exhibit avoidance behavior to reduce collision risks. In addition to these visualizations, we report quantitative metrics including ADE, FDE and AAD for all models. The results show that TSLNet achieves the lowest ADE and the smallest average avoidance angle deviation, closely followed by ViViT, whereas other models exhibit substantially larger errors. These quantitative results, together with the visualizations, confirm that only TSLNet and ViViT effectively capture realistic avoidance behavior, demonstrating the robustness of TSLNet under challenging interactive scenarios.

## 4 Discussion

This study introduced TSLNet, a new multi-task learning framework designed for simultaneous pedestrian tracking and behavior recognition. The experimental results presented in the previous sections demonstrate that TSLNet significantly outperforms existing baseline models in both pedestrian tracking and behavior recognition tasks across multiple public datasets and a self-built dataset. Specifically, TSLNet achieved higher Accuracy, Precision, Recall, F1-Score, and Mean Average Precision (mAP) in behavior recognition, as well as superior Multiple Object Tracking Accuracy (MOTA) and ID F1 Score (IDF1) in pedestrian tracking. These improvements were not only substantial but also statistically significant, as confirmed by the paired t-tests conducted.

The enhanced performance of TSLNet can be attributed to its robust architectural components, including the dual-stream Convolutional Neural Networks (CNNs), Long Short-Term Memory (LSTM) modules, and multi-head attention mechanisms. The dual-stream CNNs effectively capture spatial and temporal features separately, allowing the model to comprehend both static and dynamic aspects of pedestrian behavior. The integration of LSTM modules facilitates the modeling of temporal dependencies, which is crucial for accurately predicting future actions based on historical data. Moreover, the multi-head attention mechanism enhances the model’s ability to focus on relevant features across different time steps, thereby improving the precision and recall rates.

However, despite its impressive performance, TSLNet has certain limitations. TSLNet requires substantial computational resources, including higher GPU memory and increased CPU utilization. This complexity may limit its deployment in resource-constrained environments or on edge devices where computational power is limited. Therefore, to improve the inference efficiency of TSLNet in practical applications, we discuss several concrete strategies for model lightweighting. First, quantization can be applied by compressing the model weights and activations from 32-bit floating-point to 8-bit integer representation. Quantization can be performed either on a per-layer or per-channel basis to balance accuracy and inference speed. In addition, quantization-aware training (QAT) can be employed to further reduce potential accuracy loss. Second, pruning can be used to remove redundant channels in convolutional or fully connected layers, based on weight magnitude or importance scores. Structured pruning is particularly suitable for achieving actual speedup on hardware. Finally, knowledge distillation can train a lightweight student network to mimic the output distributions or intermediate feature representations of the original TSLNet. The student model can reduce the number of channels, layers, or simplify attention modules to achieve computational savings while maintaining performance. Although these strategies have not been experimentally implemented in this work, they provide actionable design directions and offer promising avenues for optimizing TSLNet deployment in resource-constrained scenarios.

With the widespread application of video surveillance systems in public safety and behavior analysis, ethical issues have become increasingly important. In particular, concerns regarding personal privacy protection, data usage consent, and the potential societal impact of model deployment must be carefully considered in practical applications. This study emphasizes that data collection and processing should comply with relevant laws and regulations, and measures such as anonymization and privacy protection should be implemented to minimize risks to individual privacy.

Regarding future work, we plan to further expand the multi-task capabilities of TSLNet, for example, applying it to anomaly behavior detection and dangerous behavior prediction scenarios. This can enhance the model’s applicability in complex environments and provide richer functionality for practical deployment. Additionally, exploring lightweight optimization of the model in resource-constrained environments and multi-modal data fusion represents promising directions for further research.

## 5 Conclusion

In conclusion, TSLNet represents a significant advancement in the field of video analysis, offering a powerful tool for enhancing pedestrian tracking and behavior recognition. Its high performance and comprehensive feature extraction capabilities make it a promising solution for applications demanding high precision and reliability. As technology continues to evolve, ongoing refinements and optimizations of TSLNet will be essential to fully realize its potential and ensure its effectiveness in dynamic and diverse real-world environments.

## Data Availability

The raw data supporting the conclusions of this article will be made available by the authors, without undue reservation.
